# The Adult Repetitive Behaviours Questionnaire-2 (RBQ-2A): A Self-Report Measure of Restricted and Repetitive Behaviours

**DOI:** 10.1007/s10803-015-2514-6

**Published:** 2015-07-09

**Authors:** Sarah L. Barrett, Mirko Uljarević, Emma K. Baker, Amanda L. Richdale, Catherine R. G. Jones, Susan R. Leekam

**Affiliations:** Wales Autism Research Centre, School of Psychology, Cardiff University, Tower Building, Park Place, Cardiff, CF10 3AT UK; Olga Tennison Autism Research Centre, Cooperative Research Centre for Living with Autism Spectrum Disorders (Autism CRC), School of Psychology and Public Health, La Trobe University, Bundoora, 3086 Australia; Olga Tennsion Autism Research Centre, School of Psychology and Public Health, La Trobe University, Melbourne, Australia

**Keywords:** Repetitive behaviours, Adults, Questionnaire, Autism, Principal components analysis

## Abstract

In two studies we developed and tested a new self-report measure of restricted and repetitive behaviours (RRB) suitable for adults. In Study 1, The Repetitive Behaviours Questionnaire-2 for adults (RBQ-2A) was completed by a sample of 163 neurotypical adults. Principal components analysis revealed two components: Repetitive Motor Behaviours and Insistence on Sameness. In Study 2, the mean RBQ-2A scores of a group of adults with autism spectrum disorder (ASD; *N* = 29) were compared to an adult neurotypical group (*N* = 37). The ASD sample had significantly higher total and subscale scores. These results indicate that the RBQ-2A has utility as a self-report questionnaire measure of RRBs suitable for adults, with potential clinical application.

## Introduction

Restricted and repetitive behaviours (RRBs) form one of the core diagnostic criteria for autism spectrum disorder (ASD; American Psychiatric Association 2013; World Health Organization 1993). This class of behaviours, driven by a desire for sameness and dislike of change (Kanner 1943), includes a wide range of motor and sensory behaviours and restricted activities that are highly frequent in their repetition and invariant in their manifestation. These behaviours are also found in neurotypical (NT) individuals and those with other developmental disorders and neuropsychological conditions (for reviews see Langen et al. 2011; Leekam et al. 2011).

Caregiver interviews and questionnaires are the most frequently used measures of RRBs. Observation measures, while effective for measuring motor and sensory behaviours, may be less sensitive for measuring less frequent restricted behaviours (e.g., Harrop et al. 2014; Honey et al. 2012). Factor analytic studies of RRBs using caregiver interviews and questionnaires have identified two sub-groups; one comprising repetitive sensory and motor behaviours such as hand flapping and rocking (RSMB), and the other comprising more abstract behaviours such as routines and circumscribed interests, which are collectively referred to as insistence on sameness (IS). This binary grouping has been found in as many as eleven previous studies of individuals with ASD (e.g., Bishop et al. 2006; Bishop et al. 2013; Cuccaro et al. 2003; Georgiades et al. 2010; Lidstone et al. 2014; Mooney et al. 2009; Papageorgiou et al. 2008; Richler et al. 2007; Richler et al. 2010; Shao et al. 2003; Szatmari et al. 2006) and in studies of NT children (e.g., Evans et al. 1997; Leekam et al. 2007b).

However, other studies have identified alternative solutions ranging from three to five different factors (e.g., Bishop et al. 2013; Honey et al. 2008; Lam and Aman 2007; Lam et al. 2008; Mirenda et al. 2010). Such differences may be due to the use of RRB measures that are different in terms of their scope and format, such as the Repetitive Behaviour Scale-Revised (RBS-R; Bodfish et al. 1999) and the Autism Diagnostic Interview-Revised (ADI-R; Lord et al. 1994). For example, the RBS-R is a questionnaire comprising questions about self-injurious behaviours, which may form a separate factor (e.g., Bishop et al. 2013; Mirenda et al. 2010). On the other hand, the ADI-R is an interview that has been reported to undersample RRBs (Lam et al. 2008).

In contrast to an extensive literature on RRBs in children with ASD and in children with neurotypical development, there is limited research on RRBs in adulthood. Some factor analysis studies of RRBs in ASD have included adults in their samples (e.g., Cuccaro et al. 2003; Georgiades et al. 2010; Lam et al. 2008; Papageorgiou et al. 2008; Shao et al. 2003). However, conclusions from these studies about RRBs in adults are limited; either because the samples span a limited age range, or because the adult samples were not separated from the child samples in the analysis. A minority of studies of ASD that have directly compared RRB symptoms in adults with those in children have found lower levels of RRBs in adults than in children (Esbensen et al. 2009; Fecteau et al. 2003; Piven et al. 1995). This pattern remains the same across the subtypes of RRBs and is consistent across gender and intellectual disability (ID), with the exception that motor stereotypies do not reduce as much over time in adults with comorbid ID (Esbensen et al. 2009). These findings indicate that RRBs in adulthood may present differently than in childhood, which has implications for clinical practice and research.

Caregiver-report methods such as the RBS-R are suitable for use with adults. However, certain items may not be applicable, such as items related to play behaviours and toys. Furthermore, once an adult leaves home caregivers may not be able to report as accurately on their behaviours. Currently there are few self-report measures of RRBs available that are suitable for adults. While there is a self-report interview of obsessive-compulsive symptoms, the Yale-Brown Obsessive-Compulsive Scale (YBOCS; Goodman et al. 1989), to our knowledge there are no published self-report measures for the full range of RRBs relevant to the diagnosis of ASD. RRBs and obsessive-compulsive behaviours overlap but they do not capture the same construct. For example, the YBOCS includes questions about intrusive imagery, which is not a feature of RRBs. The Autism-Spectrum Quotient (AQ; Baron-Cohen et al. 2001) is a measure of autistic traits that includes items related to RRBs (e.g., *It does not upset me if my daily routine is disturbed*). However, factor analyses suggest that the AQ does not provide an adequate or reliable assessment of RRBs in NT adults (Kloosterman et al. 2011; Lau et al. 2013).

Therefore, in the current study we adapted and tested a parent-report questionnaire to provide the first self-report RRB questionnaire suitable for adults with ASD. Following the pattern of previous research on RRBs in both NT and ASD children, we assessed the questionnaire initially in NT adults and then applied it to an ASD sample. In contrast to research on RRBs in NT children, research on the full range of RRBs in NT adults is sparse and limited to particular behaviours such as pre-sleep rituals and transition objects (Markt and Johnson 1993). Therefore, new evidence on self-reported RRBs in NT individuals will enable comparison with evidence from adults with ASD, providing further insight into the presentation of these behaviours in adults both with and without ASD. Beyond comparison purposes, it would be useful to understand the pattern of RRBs in an adult NT population. Furthermore, given the increasing need by clinicians for briefer and more streamlined methods for diagnosis, a self-report format for eliciting information on RRBs in able adults has application as a supplement to add information to other diagnostic methods.

The Repetitive Behaviour Questionnaire-2 (RBQ-2; Leekam et al. 2007b) is a twenty item questionnaire, with items directly derived from a standardised clinical interview tool, the Diagnostic Interview for Social and Communication Disorders (DISCO; Wing et al. 2002). The DISCO has good inter-rater reliability and discriminant validity (Leekam et al. 2002; Maljaars et al. 2012; Nygren et al. 2009) and shows strong agreement with outputs from the ADI-R (Nygren et al. 2009) and Autism Diagnostic Observation Schedule (ADOS; Maljaars et al. 2012). Items from the DISCO and converging items from a semi-structured interview, the Repetitive Behaviours Interview (RBI; Turner 1996, unpublished doctoral thesis), were adapted into a questionnaire measure, the RBQ-2. The RBQ-2 includes 20 RRB items; 13 identical items taken from both interviews, five items unique to the DISCO and two unique to the RBI (Leekam et al. 2007b).

The RBQ-2 was originally tested in a large sample (*N* = 679) of NT two-year-olds (Leekam et al. 2007b). There was satisfactory endorsement of all RRBs, and exploratory factor analysis supported both a four- and two-factor solution. The four-factor solution comprised: repetitive motor movements, adherence to routine, restricted interests, and unusual sensory interests. The two-factor solution comprised RSMB, which corresponded to repetitive motor movements and unusual sensory interests, and IS, which corresponded to adherence to routine and restricted interests. The reliability and validity of the RBQ-2 has since been further supported in NT 15-month-olds (Arnott et al. 2010). Finally, the RBQ-2 has also been assessed in children and adolescents with ASD (*N* = 120; Lidstone et al. 2014). Reflecting Leekam et al.’s (2007b) findings, principal components analysis (PCA) for this ASD sample also resulted in two components: RSMB and IS, with good internal consistency across the whole scale (*α* = .86) and for both RSMB and IS (*α* = .79, *α* = .83, respectively). Overall, the similarity of results across studies, satisfactory endorsement of items and good internal consistency support the construct validity of the RBQ-2 in children.

For the current research, the RBQ-2 was adapted into an adult self-report questionnaire, the Adult RBQ-2 (RBQ-2A). As the RBQ-2A has been adapted into a self-report measure, it is only accessible to participants with sufficient cognitive resources and verbal ability to complete the questionnaire. Approximately half of children with ASD are reported to have an IQ in the average range (e.g., Charman et al. 2011), suggesting that the RBQ-2A will be accessible to a similar proportion of adults with ASD. However, given the hetereogeneity of ASD, this represents a potential limitation of the RBQ-2A. Nevertheless, there is still need for a self-report measure of RRB for adults with ASD for the reasons discussed earlier.

Two studies are reported here. In Study 1, to build upon the findings with NT children, the RBQ-2A was administered to a group of NT young adults. Consistent with the majority of previous factor analytic research, PCA analysis was used to identify factors and the internal consistency of the measure was also assessed. In Study 2, the RBQ-2A was administered to a sample of NT adults with a broader age range and a sample with an ASD diagnosis to establish whether the RRB scores in those with a diagnosis of ASD was higher than those in the NT group. A secondary aim of Study 2 was to assess whether the subscales derived from Study 1 were reliable in a more representative sample.

## Study One

For Study 1, the RBQ-2A was administered to a NT university student sample and the structure of the RBQ-2A assessed using principal components analysis (PCA). It was expected that two components would emerge, as this structure is the most consistent finding, and that they would be broadly similar to that found in the original RBQ-2 (Leekam et al. 2007b; Lidstone et al. 2014). Its internal consistency was also assessed using Cronbach’s alpha. Finally, we also included the Autism-Spectrum Quotient (AQ; Baron-Cohen, et al. 2001), which has been used widely to assess the presence of a variety of autistic traits in the general population (e.g., Hurst, Mitchell, Kimbrel, Kwapil et al. 2007; Kloosterman et al. 2011; Stewart and Austin 2009). It was expected that scores on the RBQ-2A would be significantly correlated with scores on the AQ.

### Method

#### Participants

There were 163 UK university students (95 female, 67 male, 1 unreported) recruited, aged between 18 and 50 years (*M* = 21.32 years, *SD* = 4.67). Participants were recruited via the university and social media. Psychology undergraduates (*N* = 120) received course credits in exchange for participation. Two participants scored at or above the clinical cut-off of 32 on the AQ and were removed from further analyses, resulting in a new sample (*N* = 161) comprising 95 women, 65 men (1 unreported) with a mean age of 21.28 years (*SD* = 4.69). The majority (*N* = 136) were aged18–22 years.

#### Materials

The Adult Repetitive Behaviours Questionnaire-2 (RBQ-2A): The original RBQ-2 caregiver questionnaire was first adapted so that it could be used by adults in a self-report format. The original RBQ-2 is sub-divided into five sections as shown in Table 1. For three sections (corresponding to items 1–6 and 13–19; responses are given on a 4-point scale, corresponding to *never or rarely* (1), *mild or occasional*/*one or more times daily* (2), *marked or notable*/*15 or more times daily* (3) and *serious or severe*/*30 or more times daily* (4). The remaining items (7 to 12 and item 20) are answered on a 3-point scale (see Table 1 for the response options for each item). In previous studies, the responses to these items are collapsed into a 3-point scale to make means and standard deviations (SDs) comparable across all items. The RBQ-2 may be scored in terms of total mean score (ranging from 1 to 3).

**Table 1:** Study 1: frequencies, percentages, means and standard deviations of neurotypical participants’ responses to all twenty Adult Repetitive Behaviour Questionnaire-2 items (*N* = 161)

Section 1	Never or rarely	One or more times daily	15 or more times daily	Mean (SD)
Do you	1	2	3	
Like to arrange items in rows or patterns?	90 (55.9 %)	69 (42.9 %)	2 (1.2 %)	1.45 (.52)
Repetitively fiddle with items? (e.g. spin, twiddle, bang, tap, twist, or flick anything repeatedly?*	31 (19.4 %)	70 (43.8 %)	59 (36.9 %)	2.18 (.73)
Spin yourself around and around?**	121 (76.1 %)	33 (20.8 %)	5 (3.1 %)	1.27 (.51)
Rock backwards and forwards, or side to side, either when sitting or when standing?***	83 (52.5 %)	53 (33.5 %)	22 (13.9 %)	1.61 (.72)
Pace or move around repetitively (e.g. walk to and fro across a room, or around the same path in the garden?)*	99 (61.9 %)	49 (30.6 %)	12 (7.5 %)	1.46 (.63)

Section 2	Never or rarely	Mild or occasional	Marked or notable	Mean (SD)
Do you	1	2	3	
Make repetitive hand and/or finger movements? (e.g. flap, wave, or flick your hands or fingers repetitively?)	65 (40.4 %)	59 (36.6 %)	37 (23 %)	1.83 (.78)
Have a fascination with specific objects (e.g. trains, road signs, or other things?)	120 (74.5 %)	39 (24.2 %)	2 (1.2 %)	1.27 (.47)
Like to look at objects from particular or unusual angles?*	120 (75.0 %)	34 (21.3 %)	6 (3.8 %)	1.29 (.53)
Have a special interest in the smell of people or objects?	125 (77.6 %)	27 (16.8 %)	9 (5.6 %)	1.28 (.56)
Have a special interest in the feel of different surfaces?*	97 (60.6 %)	54 (33.8 %)	9 (5.6 %)	1.45 (.60)
Have any special objects you like to carry around?*	124 (77.5 %)	30 (18.8 %)	6 (3.8 %)	1.26 (.52)
Collect or hoard items of any sort?	106 (65.8 %)	48 (29.8 %)	7 (4.3 %)	1.39 (.57)

Section 3	Never or rarely	Mild or occasional (does not affect others)	Marked or notable (occasionally affects others)	Mean (SD)
Do you	1	2	3	
Insist on things at home remaining the same? (e.g. furniture staying in the same place, things being kept in certain places, or arranged in certain ways?)**	74 (46.5 %)	68 (42.8 %)	17 (10.7 %)	1.64 (.67)
Get upset about minor changes to objects (e.g. flecks of dirt on your clothes, minor scratches on objects?)	86 (53.4 %)	60 (37.3 %)	15 (9.3 %)	1.56 (.66)
Insist that aspects of daily routine must remain the same?	96 (59.6 %)	54 (33.5 %)	11 (6.8 %)	1.47 (.62)
Insist on doing things in a certain way or re-doing things until they “just right”?	72 (44.7 %)	70 (43.5 %)	19 (11.8 %)	1.67 (.68)

Section 4	Never or rarely	Mild or occasional (not entirely resistant to change or new things)	Marked or notable (will tolerate changes when necessary)	Mean (SD)
Do you	1	2	3	
Play the same music, game or video, or read the same book repeatedly?*	57 (35.6 %)	74 (46.3 %)	29 (18.1 %)	1.83 (.71)
Insist on wearing the same clothes or refuse to wear new clothes?*	131 (81.9 %)	24 (15.0 %)	5 (3.1 %)	1.21 (.48)
Insist on eating the same foods, or a very small range of foods, at every meal?*	118 (73.8 %)	35 (21.9 %)	7 (4.4 %)	1.31 (.55)

Section 5	A range of different and flexible self-chosen activities	Some varied and flexible interests but commonly choose the same activities	Almost always choose from a restricted range of repetitive activities	Mean (SD)
	1	2	3	
What sort of activity will you choose if you are left to occupy yourself?	53 (32.9 %)	91 (56.5 %)	17 (10.6 %)	1.78 (.62)

* *N* = 160; ** *N* = 159; *** *N* = 158; Percentages given as valid percentages

The original RBQ-2 was adapted for use with adults by editing the phrasing of questions. The phrase “*does your child*” at the beginning of questions 1 to 19 was changed to “*do you*”, and question 20 was changed from “*what sort of activity will your child choose if they are left to occupy themselves?*” to “*what sort of activity will you choose if you are left to occupy yourself?*” Child-specific words such as *toys* were either replaced with other similar concept words (e.g., *objects*) or removed entirely from items 1, 2, 11 and 14. This edited version of the RBQ-2 was piloted with a small group (*N* = 16; 8 male) with a mean age of 26.26 years (*SD* = 9.09 years). Even though the questionnaire was only edited minimally, there was good internal consistency for the scale (*α* = .73), a range of responses (*M* = 1.51, *SD* = .24), and few participants reported any difficulties with the questionnaire. This version of the RBQ-2A was not edited further before being administered to the present sample.

The Autism-Spectrum Quotient (AQ): The AQ (Baron-Cohen et al. 2001) is a self-report questionnaire assessing the presence of autistic features in the general population, (Baron-Cohen et al. 2001). It comprises 50 statements based on the original triad of impairments (social interaction, social communication and imagination; Wing and Gould 1979), and other aspects of cognitive processing in ASD. Each participant receives a score out of 50, with higher scores indicating greater endorsement of autistic traits. In most studies, a score of 32 is considered the clinical cut-off for ASD as according to the original paper (Baron-Cohen et al. 2001). Later research has recommended a more stringent cut-off of 26 (Woodbury-Smith et al. 2005). Here we chose to implement the the original cut-off score of 32 in order to preserve sample size and variation.

#### Procedure, Data Screening and Statistical Analyses

Ethical approval was obtained from the university’s School of Psychology Ethics Committee, and informed consent was obtained from the participants before they completed the questionnaires. The online questionnaires were presented on Google Documents, with the RBQ-2A presented first followed by the AQ. The data were analysed using SPSS 20.

To maintain consistency with previous research (Leekam et al. 2007b; Lidstone et al. 2014) item 20 was included in the total score but was removed before factor analysis as its response scale differs to the other items (Leekam et al. 2007b; see Table 1). In addition, for the factor analysis stage only, items were removed before analysis if 80% or more of the sample responded *never or rarely*; this resulted in item 18 (clothing) being excluded from the analysis. Responses were scored in line with previous studies using a mean severity score (range 1–3).

An initial PCA was run to obtain Eigenvalues for each component. Components were extracted using parallel analysis (Horn 1965) with the Monte Carlo PCA for Parallel Analysis program. This is a more stringent criterion for component extraction than Kaiser’s criterion or the Scree plot (Tabachnick and Fidell 2014). Due to the small sample size, we employed a relatively high cut-off for determining what items were meaningfully associated with a component; here the cut-off was .4 (as in Honey et al. 2008; Szatmari et al. 2006) and cross-loading items were excluded. The internal consistency for the whole scale and each of the resultant components was also assessed by calculating Cronbach’s alpha (*α*) values.

### Results

Table 1 shows the endorsement, mean total scores and *SD* for all 20 RBQ-2A items. For every item, at least 14.9 % of the sample endorsed *mild or occasional* or higher. The mean total score for all RBQ-2A items for the sample (*N* = 161) ranged from 1 to 2.55 (*M* = 1.51, *SD* = .30). The internal consistency of the whole scale was good (Cronbach’s *α* = .83).

#### Principal Components Analysis (PCA)

Several participants had missing data (*N* = 13) across the 18 RBQ-2A items being included in the analysis. A Missing Value Analysis was conducted on the dataset for these 18 items. As Little’s Missing Completely at Random test was non-significant and the percentage of participants with missing data was small (8.07 %) it was appropriate to exclude these participants from the analysis (Tabachnick and Fidell 2014).

The final sample used for the PCA comprised 148 participants (87 female, 60 male, 1 unreported) with a mean age of 21.3 years (*SD* = 4.79; 22 participants were 23 years or older) and a mean total RBQ-2A score of 1.52 (*SD* = .30). The mean scores of the participants were significantly positively skewed, as found in the analysis of other RRB questionnaires. Age was also positively skewed with five outliers. However, age was not significantly correlated with RBQ-2A score (*r*_*s*_ = .01, *p* = .88). Therefore, to preserve variation and sample size these five outliers remained in the PCA. Mean total AQ score was 13.82 (*SD* = 5.99), which was normally distributed.

Initial screening indicated that the assumptions of sampling adequacy (Kaiser-Meyer-Olkin = .79), multicollinearity and factorability were all met. The initial PCA solution resulted in six components with eigenvalues greater than one, explaining 62.03% of the variance. Parallel analysis indicated that two components should be retained, so the analysis was re-run specifying two components. When running the PCA with oblique rotation (Direct Oblimin), the correlation between the two components was above .32, indicating oblique rotation should be retained (Tabachnick and Fidell 2014).

This solution explained 35.83 % of the variance after Direct Oblimin rotation. Table 2 shows the rotated item loadings (from the pattern matrix), percentage of variance explained and Cronbach’s alpha values for each of the components. There were no cross-loading items, but four items did not load sufficiently on to either component. The first component corresponds approximately to RSMB but with no sensory items; therefore it is named Repetitive Motor Behaviours (RMB). The second corresponds to insistence on sameness IS as in previous research. The internal consistency (Cronbach’s alpha) of both scales is good (>.70).

**Table 2:** Study 1: pattern matrix for principal components analysis of neurotypical data, percentage of variance explained, internal consistency and descriptive statistics for each component

Rotated item loadings	Component 1	Component 2
	Repetitive motor behaviour (RMB)	Insistence on sameness (IS)
Like to arrange items in rows or patterns?	**.447**	.140
Repetitively fiddle with items?	**.611**	.054
Spin yourself around and around?	**.713**	−.084
Rock backwards and forwards, or side to side, either when sitting or when standing?	**.871**	−.207
Pace or move around repetitively	**.718**	−.067
Make repetitive hand and/or finger movements?	**.686**	.029
Have a fascination with specific objects?	.390	.261
Like to look at objects from particular or unusual angles?	.233	.218
Have a special interest in the smell of people or objects?	.178	.308
Have a special interest in the feel of different surfaces?	.383	.250
Have any special objects you like to carry round?	.181	**.424**
Collect or hoard items of any sort?	−.078	**.503**
Insist on things at home remaining the same?	.001	**.702**
Get upset about minor changes to objects?	−.103	**.695**
Insist that aspects of daily routine must remain the same?	−.102	**.716**
Insist on doing things in a certain way or re-doing things until they are “just right”?	.184	**.505**
Play the same music, game or video, or read the same book repeatedly?	.164	**.507**
Insist on eating the same foods, or a very small range of foods, at every meal?	−.041	**.438**
Percentage of variance explained:	25.67 %	10.16 %
Cronbach’s alpha (*α*):	.78	.73
Mean (SD)	1.65 (.46)	1.54 (.37)
Median (IQR)	1.50 (.67)	1.50 (.47)

Bold denotes items that load on each factor (item loading > .4)

#### Subsidiary Analyses

For the following analyses, non-parametric statistics were used where the data were not normally distributed. Firstly, the within-participant difference between sub-scale scores was assessed. Mean total scores on both RMB and IS were significantly positively skewed, although there were no outliers. Table 2 shows the means, SDs, medians and interquartile ranges (IQRs) of the two components. There was a significant correlation between the two components (*r*_*s*_ = .35, *p* < .001). A Wilcoxon’s signed ranks test indicated that participants scored significantly more highly on RMB than IS (*Z* = −2.79, *p* = .005). These results indicate that there is a small but significant difference between sub-scale scores.

Mean total score on the RBQ-2A was significantly and positively correlated with mean total score on the AQ (*r*_*s*_ = .57, *p* < .001), which remained significant when removing two outliers on RBQ-2A (*r*_*s*_ = .56, *p* < .001). Mean total AQ score was also significantly positively correlated with both RMB (*r*_*s*_ = .35, *p* < .001) and IS (*r*_*s*_ = .54, *p* < .001).

## Discussion

The aim of Study 1 was to develop and test the RBQ-2A as a self-report measure of RRBs in NT adults. An existing parent report measure of RRBs, the RBQ-2 (Leekam et al. 2007b), was adapted into an adult self-report measure and administered to a university student sample. PCA resulted in a two-component structure, one comprising motor behaviours, RMB, and the other behaviours related to routines and a preference for sameness, IS. As predicted, scores on the RBQ-2A were also correlated with another measure of autistic traits, the AQ (Baron-Cohen et al. 2001).

The first component, RMB, is similar to RSMB found in previous research with the RBQ-2 (e.g., Leekam et al. 2007b; Lidstone et al. 2014). Five of the six RMB items consistently load onto the factor that in previous research included motor and sensory items (RSMB), the exception being item one, *arranging objects*. The major difference between RMB found here and RSMB in previous research is the lack of sensory items loading onto this component. The second component corresponded to IS. This result was more comparable to previous research using the RBQ-2 in an ASD sample, with five items (13–17) loading in the same way as in Lidstone et al.’s (Lidstone et al. 2014) study.

In summary, the components yielded by the present PCA are similar to previous research with NT children and autistic children using the RBQ-2, with the exception of sensory items. Items two to six load onto RSMB in the child version of the questionnaire (Leekam et al. 2007b; Lidstone et al. 2014) and RMB in the present study, and items 13 to 17 load onto IS across all three studies, supporting the construct validity of the questionnaire.

The most probable reason for the difference between the present PCA solution and previous research is that the present sample comprised NT adults whereas previous research examined NT children (Leekam et al. 2007b) and children and adolescents with ASD (Lidstone et al. 2014). Certain types of behaviours may be associated with younger children or children with ASD rather than NT adults. For example, mean scores on items 3 (spinning) and 11 (carrying around objects) were higher in NT children (Arnott et al. 2010; Leekam et al. 2007b) than in the present study. Moreover, autistic individuals show higher levels of sensory symptoms than NT individuals (e.g., Ben-Sasson et al. 2009; Kern et al. 2006; Leekam et al. 2007a; Rogers and Ozonoff 2005) and these items were not well endorsed by the present sample.

The different loading of certain items may also reflect the fact that certain behaviours do not clearly fall into one particular category. For example, eating a small range of foods (item 19) formed part of IS in the present study but has previously loaded on to RSMB (Lidstone et al. 2014) as well as IS (Leekam et al. 2007b); eating a small range of food may be a result of sensory issues or insistence on sameness and is therefore conceptually related to both subscales.

There are some limitations in terms of the sample. Firstly, the sample comprised only university students and is therefore limited in age and IQ distribution. Second, it might be considered that the size of the sample is relatively small for PCA. However, the literature is equivocal regarding the appropriate sample size for PCA and factor analysis (e.g., Tabachnick and Fidell 2014; Williams et al. 2010) and the assumptions for PCA were met. Therefore, the data were deemed suitable for analysis. Overall, the results of Study 1 support the construct validity of the RBQ-2A and suggest that it is useful as a self-report questionnaire in an adult population.

## Study Two

In Study 1, the sample comprised young NT adults. In Study 2, the RBQ-2A was administered to older NT adults and to adults with ASD. It was hypothesised that the ASD sample would score significantly higher than the NT group on the RBQ-2A. This study also explored the reliability of the subscales found in Study 1 in a more representative NT sample. As all RBQ-2A items were administered, including the sensory items that did not load on to the PCA in Study 1, this study also offered the opportunity to examine the RBQ-2A sensory scores in ASD as compared with NT adults.

### Method

#### Participants

Data were collected from two groups of adults who were participating in a larger study of adults with ASD being carried out in Australia. All participants completed a screening questionnaire that included first language, ASD diagnosis or family history of ASD, comorbid diagnoses, other medical and health related diagnoses, employment and marital status, living arrangements and medication. To be accepted into the study, ASD adults needed to have a confirmed clinical diagnosis of ASD (clinical reports were provided), while NT adults all had an AQ score <26 (Woodbury-Smith et al. 2005). Any individual with a diagnosis of schizophrenia was also excluded from the study. Furthermore, NT adults were excluded if they had a first degree relative with ASD, or if they had an anxiety or mood disorder. All participants had at least average intellectual ability (IQ > 80) and the NT and ASD samples were group-wise matched for Performance IQ (PIQ), Verbal IQ (VIQ) and Full Scale IQ (FSIQ), as shown in Table 3.

**Table 3:** Study 2: verbal IQ, performance IQ, and full scale IQ ranges, mean scores and standard deviations (SDs) and their correlations with mean Adult Repetitive Behaviour Questionnaire-2 total scores for both neurotypical (NT) and autism spectrum disorder (ASD) groups

	NT				ASD			
	Mean (SD)	Range	N	Correlation with RBQ-2A	Mean (SD)	Range	N	Correlation with RBQ-2A
Verbal IQ	115.06 (8.77)	97–130	34	*r*_*s*_ = −.17, *p* = .34	118.92 (11.64)	95–143	25	*r* = .07, *p* = .73
Performance IQ	115.74 (9.93)	96–134	34	*r*_*s*_ = −.06, *p* = .75	116.48 (13.68)	89–150	25	*r* = −.07, *p* = .74
Full scale IQ	117.44 (8.61)	99–133	34	*r*_*s*_ =−.10, *p* = .59	120.22 (12.08)	96–145	27	*r* = .03, *p* = .90

The ASD group (*N* = 29) comprised 15 women and 14 men aged 21.86 to 44.23 years (*M* = 34.27, *SD* = 6.29). The NT group (*N* = 37) comprised 23 women and 14 men aged 21.90 to 43.32 years (*M* = 30.75, *SD* = 6.21), with a mean AQ score of 11.78 (*SD* = 4.41). For the NT group, 48.6 % were employed on a full time basis, 24.3 % worked part-time, 24.3 % were students and 2.7 % were unemployed. For the ASD group, 27.6% were employed full time, 24.1 % worked part-time, 6.9 % were home keepers, 13.8 % were students and 27.6 % were unemployed. ASD participants were recruited through various Australian Autism Associations, the research centre’s Research Participant Registry as well as flyers displayed at clinics specialising in ASD. The NT participants were recruited primarily through the School of Psychological Science participant registry, social media, and flyers placed around the university and in the general public. This study received ethical approval from the university’s Human Ethics Committee. Informed consent was obtained for all participants.

#### Materials

As in Study 1, all participants completed the RBQ-2A and the AQ as part of an online survey comprising several different questionnaires.

As noted above, all ASD participants provided a copy of their clinical report confirming their diagnosis. They were also assessed using the Autism Diagnostic Observation Schedule-Second edition (ADOS-2). The ADOS-2 (Lord et al. 2012) is a semi-structured observation schedule that is used clinically to diagnose ASD and confirm diagnoses for research purposes. ADOS-2 data were available for 27 of the ASD group; the remaining two participants were recruited interstate and funds were not available to travel to assess them. The total ADOS-2 score ranged from 4 to 19 for this sample (*M* = 11.59, *SD* = 4.23). Six participants (21 %) did not meet the criteria for ASD according to the recently revised ADOS-2 algorithm (Lord et al. 2012), which is similar to the rate reported by Bastiaansen et al. (2011). However, when removing participants who did not meet ADOS-2 criteria for ASD from the analyses, the pattern of results did not change (with the exception of internal consistency of RMB in the ASD group falling below .70). Therefore, as these participants had a confirmed clinical diagnosis of ASD they remained in the analysis to preserve statistical power.

Twenty-three (79 %) of the ASD participants and 34 (92 %) of the NT participants completed the Wechsler Abbreviated Scale of Intelligence (WASI-II; Wechsler 2008) to gain estimates of VIQ, PIQ and FSIQ. Four of the ASD participants had recently completed the Wechsler Adult Intelligence Scale (WAIS-III) as part of their diagnostic assessment and IQ scores were obtained from their diagnostic reports. Participants who were not assessed lived interstate (2 ASD, 3 NT). No participants scored below 89 on any of the IQ measures. Participants who did not complete an IQ assessment held, or were in the process of completing, a diploma or Bachelor’s degree and thus were considered high-functioning. The means, SDs and ranges for all three IQ scores for both groups are shown in Table 3.

#### Data Screening and Statistical Analyses

As in Study 1, non-parametric tests were used for data that were not normally distributed. The ASD group was significantly older than the NT group [*t*(1, 60.26) = 5.08. *p* = .03].^1^ Therefore any group differences may be confounded by age. However, age was not significantly correlated with participants’ mean total score on the RBQ-2A in either the NT (*r*_*s*_ = .08, *p* = .65) or ASD group (*r* = −.02, *p* = .94). Welch’s t-tests, which correct for unequal sample sizes, showed no significant differences between the two groups in terms of VIQ [*t*(1, 42.8) = 1.94, *p* = .17], PIQ [*t*(1, 41.64) = .05, *p* = .82) or FSIQ (*t*(1, 45.39) = 1.02, *p* = .32]. Furthermore, mean total score on the RBQ-2A was not significantly correlated with VIQ, PIQ or FSIQ in either of the participant groups (see Table 3).

### Results

The means, SDs, medians and IQRs for RBQ-2A across the two groups are shown in Table 4. Mean RBQ-2A total scores ranged from 1 to 1.8 in the NT group and from 1.05 to 2.75 in the ASD group. Participants in the ASD group scored significantly higher on the RBQ-2A than participants in the NT group (*Z* = −5.43, *p* < .001, *r* = −.67), indicating a large effect size. No significant sex differences were found in mean RBQ-2A in either the NT or ASD group (*p* > .05). There were significant positive correlations between mean total RBQ-2A score and mean total AQ score in both ASD participants (*r*_*s*_ = .56, *p* = .002) and NT participants (*r*_*s*_ = .42, *p* = .01). Finally, the internal consistency of the RBQ-2A was good in the NT group (*α* = .73) and excellent in the ASD group (*α* = .91).

**Table 4:** Study 2: means, standard deviations (SD), medians and interquartile ranges (IQR) for the mean total RBQ-2A score and the components RMB and IS

	ASD group		NT group	
	Mean (SD)	Median (IQR)	Mean (SD)	Median (IQR)
Total RBQ-2A score	1.84 (.45)	1.90 (.78)	1.25 (.19)	1.20 (.25)
RMB score	1.59 (.45)	1.50 (.58)	1.26 (.28)	1.17 (.33)
IS score	2.04 (.55)	2.0 (1.0)	1.29 (.25)	1.25 (.25)
RSMB score	1.64 (.47)	1.60 (.70)	1.20 (.24)	1.10 (.30)

Mean scores on the two subscales identified in Study 1, RMB and IS, were calculated for all participants. The means, SDs, medians and IQRs of the mean scores on each component for the two groups are shown in Table 4. The ASD group scored significantly higher than the NT group on both RMB (*Z* = −3.32, *p* = .001, *r* = −.41) and IS (*Z* = −5.51, *p* < .001, *r* = −.68), indicating medium and large effect sizes respectively. There were no significant differences between scores on RMB and IS for the NT group (*Z* = −.90, *p* = .37). However, in the ASD group participants scored significantly lower on the RMB subscale compared to the IS subscale [*t* (28) = −5.62, *p* < .001]. There were also no significant sex differences in either of the subscales in both groups (*p* >.05). In the ASD group, the internal consistency was good for both RMB (*α* = .75) and IS (*α* = .87). For the NT group internal consistency was acceptable for RMB (*α* = .65) but poor for IS (*α* = .55). In addition, RMB and IS were significantly correlated in the ASD group (*r* = .64, *p* < .001) but not in the NT group (*r*_*s*_ = .15, *p* = .37).

The subscales of the RBQ-2A as identified from Study 1 exclude sensory items (items 7, 8, 9 and 10). As sensory atypicalities are a behavioural feature of ASD, an RSMB variable was created, comprising the RMB and sensory items (items 1–10, see Table 1). The mean RSMB score of the NT group was 1.20 (*SD* = .24; *α* = .76) and the mean RSMB score for the ASD group was 1.64 (*SD* = .47; *α* = .85). The medians and IQRs are displayed in Table 4. The ASD group scored higher than the NT group in terms of RSMB (*Z* = −4.20, *p* < .001, *r* = −.52), with a large effect size. There was no significant within-participant difference between RSMB and IS for the NT group (*Z* = −1.68, *p* = .09) but there was for the ASD group (*t*(28) = −5.11, *p* < .001). Again there were no significant sex differences in terms of RSMB in either group (*p* > .05).

#### Subsidiary Analyses

The RBQ-2A scores of the Study 1 sample were also compared to the NT group from Study 2. In order to create matched groups, only the older participants from each group (aged 23 years and older) and those with an AQ score <26 were selected. This resulted in two NT groups: one from Study 1 (*N* = 20) and one from Study 2 (*N* = 34), which did not significantly differ in terms of age (*Z* = −1.68, *p* = .09) or AQ score [*t*(1, 36.77) = 3.54, *p* = .07]. The mean age, RBQ-2A and AQ scores are displayed in Table 5. Group comparison of RBQ-2A total scores showed that the Study 1 NT group scored significantly higher on the RBQ-2A^2^ than the Study 2 NT group [*t*(1, 27.83) = 12.04, *p* = .002]. The Cronbach’s alpha of the older participants from Study 1 was good (*α* = .87), lending further support to the internal consistency of the RBQ-2A in older adults. In addition, when this Study 1 NT subgroup was compared with the ASD participants aged 23 years and older [*N* = 26; mean age = 35.64 (*SD* = 5.03); mean RBQ-2A score = 1.83 (*SD* = .44)], the ASD participants still scored significantly more highly than the Study 1 subgroup [*t*(1, 44) = 8.02, *p* = .007].

**Table 5:** Study 2: means, standard deviations (SD), medians and interquartile ranges (IQR) for the mean total RBQ-2A and components scores for the two NT groups

	Study 1 NT group		Study 2 NT group	
	Mean (SD)	Median (IQR)	Mean (SD)	Median (IQR)
Age (years)	30.0 (8.59)	25.51 (12.04)	31.52 (5.88)	30.01 (10.12)
Mean total RBQ-2A score	1.51 (.33)	1.40 (.39)	1.24 (.17)	1.20 (.25)
Total AQ score	14.4 (5.09)	–	11.79 (4.60)	–

### Discussion

Study 2 explored the difference in RBQ-2A scores between NT and adult ASD participants. In line with the hypothesis, participants with ASD scored significantly higher on the RBQ-2A than IQ-matched NT participants, in terms of both total score and scores on the subscales identified in Study 1. This supports the utility of the RBQ-2A as a measure of RRBs in adults with ASD as it is able to detect differences in RRBs between autistic and NT groups. Additionally, the internal consistency of the RBQ-2A was supported in this study, with the exception of the IS subscale in the NT group.

These results indicate that the RBQ-2A is able to distinguish between NT and ASD participants at a group level, as NT participants rate themselves lower on RRBs. However, this finding would be strengthened by assessing the accuracy of self-report, by testing the correlation between the RBQ-2A and another type of measure such as parent-report or observation. Some argue that individuals with ASD find introspection and reporting their symptoms difficult (e.g., Williams 2010). Nevertheless, expected group differences were detected, and the internal consistency of RBQ-2A and its sub-scales ranged from good to excellent in the ASD group, indicating that adults with ASD are able to self-report RMB and IS behaviours with accuracy.

Interestingly, while there is no significant difference between the subscales of RMB and IS in the NT sample, participants with ASD rate themselves significantly more highly on IS compared to RMB. This suggests that among older adults with ASD, reported IS behaviours are particularly high compared to RMB. This pattern was repeated when including sensory items in the RMB factor to create RSMB. In addition, the NT group scored themselves significantly lower compared to the ASD group on all three subscales. For both groups, addition of sensory items increased the internal consistency compared to RMB. For the NT group, addition of sensory items slightly reduced the mean (from 1.26 to 1.20) whereas for the ASD group, addition of sensory items increased the mean (from 1.59 to 1.64), increasing the difference between the two groups established on the RMB subscale. This reflects previous research that found autistic individuals show higher levels of sensory symptoms than NT individuals (e.g., Kern et al. 2006). Given these results, it is important to retain the sensory items in the RBQ-2A when it is administered to an ASD sample.

These results indicate that when matching the two NT groups in terms of age and total AQ score, the Study 1 university students scored more highly on the RBQ-2A than the Study 2 sample. Therefore it seems unlikely that either differences in age or AQ score account for the differences in RBQ-2A scores of both two NT groups. Although the two samples were recruited from different countries, both are Western countries, making country of origin an unlikely explanation for the difference in RBQ-2A score. A more plausible explanation might be that in Study 2, NT participants with anxiety or mood disorders were excluded, while this did not occur in Study 1. Furthermore, the participants in Study 1 were university students and it has been shown that university students have high levels of anxiety symptoms compared to the general population (e.g., Andrews and Wilding 2004; Stallman 2010). Anxiety may be related to RRB, for example rituals in university students (Markt and Johnson 1993) and in children and adolescents with ASD (e.g., Lidstone et al. 2014; Rodgers et al. 2012).

## General Discussion

Overall, these results indicate that the RBQ-2A is a useful new self-report measure for assessing RRBs in adults. Study 1 found a two-component structure in a NT university student sample that approximately corresponds to previous research using other measures of RRBs, with the exception of sensory items. Study 2, using a more representative sample of adults, found that participants with ASD score significantly more highly than IQ-matched NT participants on the RBQ-2A total and subscale scores, which would be expected from an accurate measure of RRBs. The internal consistency of the RBQ-2A and its subscales was high for adults with ASD, providing further support to its reliability as a measure of RRBs for adults on the autism spectrum. Both studies showed that RRBs are significantly associated with AQ score and support the use of the RBQ-2A as a measure of RRBs in NT adults. Subsidiary analyses in Study 2 also indicated that although the university sample in Study 1 had higher levels of RRB than the adults in Study 2, this was unlikely to be due to differences in age. Given the potential relationship between RRBs and anxiety, it can be speculated that the higher incidence of anxiety traits in university populations (Andrews and Wilding 2004; Stallman 2010), alongside the screening for significant psychopathology in the Study 2 NT sample, may have biased the Study 1 group to relatively higher scores. Further research is needed to explore both the association between psychopathology symptoms and RRBs, and whether the RBQ-2A can accurately distinguish between ASD and psychological disorders that involve high levels of RRB, such as OCD and other specific anxiety disorders.

Another unexpected finding from Study 1 is that most sensory items from the RBQ-2A did not load onto either component. Furthermore, adding sensory items to the RMB subscale for the NT group in Study 2 reduced the mean score on this subscale. These findings indicate that sensory symptoms are not common in the NT participants across both studies, whereas they are highly prevalent within the autistic population (e.g., Boyd et al. 2010; Kern et al. 2006; Leekam et al. 2007a). This may be partly explained by the fact that some of the sensory items contain references to ‘special’ interests and items, which may not be relevant for NT adults. Alternatively, it may be that the RBQ-2A simply does not capture a wide enough range of sensory behaviours, as it includes just six items from the original set of 25 items in the DISCO (items 7, 8, 9, 10, 18 and 19). A previous study of the general population found evidence of a wider range of sensory behaviours with a more detailed questionnaire, the Glasgow Sensory Profile (Robertson and Simmons 2013). This questionnaire covers seven modalities, including auditory, vestibular and proprioceptive, which are not included in the RBQ-2A. Nevertheless, the RBQ-2A was able to discriminate between the ASD and NT groups both with and without the sensory items.

There are also some important limitations to consider for the studies reported here. Although the samples across both studies include a fairly wide range of ages from 18 to 50 years old, these findings cannot be generalised to adults of an older age. Furthermore, as the RBQ-2A was adapted from a measure for children, it may be missing certain items that are applicable only to adults. As discussed in the Introduction, the RBQ-2A as a self-report measure is currently suitable only for more able adults. Therefore the RBQ-2A, and any associated findings, are only generalizable to this population. In both studies, there was significant positive correlation between the AQ and the RBQ-2A. However this correlation might be partly explained by the fact both are self-report measures. As mentioned, it is therefore important to compare the RBQ-2A with other measures of autistic traits such as interviews or informant-report questionnaires. The purpose of the RBQ-2A is to describe a profile of RRBs; it does not measure social communicative behaviours and therefore is not suitable as a stand-alone diagnostic tool for ASD, which includes both domains as necessary and essential conditions for a diagnosis.

Nevertheless, the studies presented here represent an important new contribution with the development of an adult self-report measure of RRBs, which can be used with both ASD and NT populations. The need for such a measure is indicated by the findings of both studies, which indicate that self-reported RRBs in adulthood may present slightly differently than carer-reported RRBs in childhood. Specifically, although the subtypes of RRBs remain the same, the specific behaviours that are endorsed differ. The potential clinical applications of the RBQ-2A include its use as a signposting questionnaire or as a supplement to diagnostic interviews such as the DISCO. Its utility may be especially helpful given that the AQ does not give an adequate or reliable assessment of RRBs across typical populations (e.g., Kloosterman et al. 2011; Lau et al. 2013). It may also be useful for other clinical conditions that show RRBs, such as obsessive-compulsive disorder, Gilles de la Tourette syndrome and Parkinson’s disease (Langen et al. 2011). From a research perspective, the RBQ-2A allows for the opportunity to accurately and reliably explore RRBs directly in adults both with and without ASD. Overall, these results show that the RBQ-2A is a promising self-report measure of RRBs in adults. However, further research should involve older and more diverse NT participants that include representation of a range of ethnic and SES groups, as well as a larger sample of adults with ASD. Although the RBQ-2A is a descriptive questionnaire that can only identify a profile of behaviours as perceived by self-informants, further research comparing self- and other-informant RBQ-2A questionnaires and its use with clinical interviews may help to assess how well the RBQ-2A complements and streamlines the diagnostic process in clinical practice.

## Footnotes

1 The age of the NT group was positively skewed, so square root transformation was applied to both samples for this test.

2 RBQ-2A scores were positively skewed in the Study 2 sample, so the RBQ-2A scores of both were transformed using natural logarithm for this analysis.
